# Aberrant neural networks for the recognition memory of socially relevant information in patients with schizophrenia

**DOI:** 10.1002/brb3.602

**Published:** 2016-11-22

**Authors:** Jooyoung Oh, Ji‐Won Chun, Eunseong Kim, Hae‐Jeong Park, Boreom Lee, Jae‐Jin Kim

**Affiliations:** ^1^ Department of Biomedical Science and Engineering (BMSE)Institute of Integrated Technology (IIT)Gwangju Institute of Science and Technology (GIST)GwangjuKorea; ^2^ Institute of Behavioral Science in MedicineYonsei University College of MedicineSeoulKorea; ^3^ Department of Nuclear MedicineYonsei University College of MedicineSeoulKorea; ^4^ Department of PsychiatryYonsei University College of MedicineSeoulKorea

**Keywords:** frontopolar network, functional magnetic resonance imaging, independent component analysis, language comprehension network, recognition memory, schizophrenia

## Abstract

**Introduction:**

Patients with schizophrenia exhibit several cognitive deficits, including memory impairment. Problems with recognition memory can hinder socially adaptive behavior. Previous investigations have suggested that altered activation of the frontotemporal area plays an important role in recognition memory impairment. However, the cerebral networks related to these deficits are not known. The aim of this study was to elucidate the brain networks required for recognizing socially relevant information in patients with schizophrenia performing an old–new recognition task.

**Methods:**

Sixteen patients with schizophrenia and 16 controls participated in this study. First, the subjects performed the theme‐identification task during functional magnetic resonance imaging. In this task, pictures depicting social situations were presented with three words, and the subjects were asked to select the best theme word for each picture. The subjects then performed an old–new recognition task in which they were asked to discriminate whether the presented words were old or new. Task performance and neural responses in the old–new recognition task were compared between the subject groups. An independent component analysis of the functional connectivity was performed.

**Results:**

The patients with schizophrenia exhibited decreased discriminability and increased activation of the right superior temporal gyrus compared with the controls during correct responses. Furthermore, aberrant network activities were found in the frontopolar and language comprehension networks in the patients.

**Conclusions:**

The functional connectivity analysis showed aberrant connectivity in the frontopolar and language comprehension networks in the patients with schizophrenia, and these aberrations possibly contribute to their low recognition performance and social dysfunction. These results suggest that the frontopolar and language comprehension networks are potential therapeutic targets in patients with schizophrenia.

## 1 Introduction

Schizophrenia is a major disorder in the psychiatric field. Patients with schizophrenia exhibit several cognitive deficits (Keefe & Harvey, 2012; Stip, 2006), especially deficits in memory (Aleman, Hijman, de Haan, & Kahn, 1999; Conklin, Curtis, Katsanis, & Iacono, 2000; Goldberg, Weinberger, Pliskin, Berman, & Podd, 1989; Saykin et al., 1991). These memory deficits generally begin during the earlier stages of the disorder (Dieleman, van der Veen, van Beveren, & Röder, 2015) and persist until the later stages (Nestor et al., 2007). In addition, this problem can lead to dysfunction in daily life (Ventura, Tom, Jetton, & Kern, 2013) as well as treatment failure (Heinrichs, Goldberg, Miles, & McDermid Vaz, 2008). Previous studies of the memory deficits of patients with schizophrenia have reported robust deficits in episodic memory in general (Heinrichs & Zakzanis, 1998; Toulopoulou, Rabe‐Hesketh, King, Murray, & Morris, 2003) and recognition memory (Danion, Rizzo, & Bruant, 1999; Grange, Robert, & Rizzo, 1995). These memory deficits can cause significant issues during social interactions because the acquisition and maintenance of social information aid the exhibition of adaptive behaviors and regulatory functions in social environments (Harvey & Lepage, 2014).

Many investigations have identified the neural mechanisms underlying the memory deficits observed in patients with schizophrenia (Leube et al., 2003; Manoach et al., 2000; Meyer‐Lindenberg et al., 2001). In particular, the role of the prefrontal cortex in working and episodic memory has been consistently emphasized (Achim & Lepage, 2005; Perlstein, Carter, Noll, & Cohen, 2001; Ragland et al., 2009). Patients generally have more structural and molecular abnormalities in their prefrontal cortex (Baare et al., 1999; Coyle & Tsai, 2004), and they therefore cannot effectively use cognitive control mechanisms (Ranganath, Minzenberg, & Ragland, 2008). Among the regions of the prefrontal cortex, the frontopolar regions are thought to be important in recognition memory (McDermott, Jones, Petersen, Lageman, & Roediger, 2000), the processing of key information (Azuar et al., 2014), and integration of information from the surrounding environment (Petrides & Pandya, 2006). Therefore, aberrant activation of this region can cause recognition memory deficits for socially important information.

The temporal lobe also plays an important role in the deficits of memory, including recognition memory, in patients with schizophrenia (Nestor et al., 1993; Yonelinas, Hopfinger, Buonocore, Kroll, & Baynes, 2001). The memory impairments in patients with schizophrenia are closely related to their language comprehension problems (Condray, Steinhauer, van Kammen, & Kasparek, 1996), which suggests that language comprehension areas, including the superior temporal lobe, might be important for memory processing. Furthermore, evidence that right‐hemisphere language function is critical to understand someone's communicative intent, such that the normal functioning of this region is needed to adjust to society, has been reported (Mitchell & Crow, 2005). Taken together, the language comprehension network in the temporal lobe, especially the right side, might be critical for remembering socially important information.

Previous studies examining both the prefrontal and temporal lobes have indicated hypoactivity in the prefrontal regions and compensatory hyperactivity in the temporal regions during memory processes in patients with schizophrenia (Achim & Lepage, 2005; Ragland et al., 2004, 2009). The prefrontal hypoactivity is thought to be related to a lack of strategies in the patient's retrieval process, and the temporal hyperactivity is considered evidence that familiarity assessments, rather than conscious recollections, are generally used during retrieval processes (Achim & Lepage, 2005).

The recognition of socially important information is essential because social dysfunction can be caused by the inability to effectively encode key social information into memory and later utilize it during future social interactions (Harvey & Lepage, 2014). Although many studies have investigated episodic and recognition memory in schizophrenia, few studies have used stimuli depicting social situations (Harvey, Fossati, & Lepage, 2007; Harvey & Lepage, 2014). The mechanisms underlying the deficits in the recognition memory of socially important information are not yet fully understood. Moreover, the previous studies did not analyze the brain networks. In fact, investigations of schizophrenia have increasingly focused on understanding neural networks and connectivity because schizophrenia is now considered a disconnection syndrome (Stephan, Friston, & Frith, 2009). This disconnection problem in patients seems to result from the abnormal wiring of the brain during development (Bullmore, Frangou, & Murray, 1997) and abnormal synaptic plasticity (Stephan, Baldeweg, & Friston, 2006). Taken together, the neural networks underlying the recognition of socially important information should be explored in patients with schizophrenia in order to better comprehend the mechanisms underlying the social dysfunction of these patients.

The purpose of this study was to investigate the brain activity and connectivity patterns of patients with schizophrenia during the recognition of socially important information. Considering the compensatory hyperactivity in the temporal lobes (Ragland et al., 2004), we hypothesized that the patients would show hyperactivity in the temporal regions as they attempted to perform at a level that was similar to the level of the healthy controls. In addition, we assumed that this phenomenon would be prominent during the recognition of socially important information and that hyperactivity would not be seen during the recognition of relatively inconsequential information. Furthermore, we postulated that the patients would exhibit decreased functional connectivity in the frontopolar and language comprehension networks.

## 2 Materials and Methods

### 2.1 Participants

Sixteen patients with schizophrenia and 16 healthy controls who were also examined in our previous study (Oh et al., 2015) participated in this study. Age did not differ between the patient and control groups. However, the two groups differed significantly for the number of years of education and IQ. The short form of the Korean‐Wechsler Adult Intelligence Scale was used to evaluate IQ (Yeom, Park, Oh, & Lee, 1992). The patients' symptom severities were assessed with the Positive and Negative Syndrome Scale (Kay, Flszbein, & Opfer, 1987). All of the patients were taking at least one antipsychotic medication, and their symptoms and medication doses were stable at the time of the study. Even though many of the patients did not have jobs, every patient was able to regularly visit the hospital to maintain their medications. Of the 16 patients with schizophrenia, 14 patients were the paranoid subtype and two patients were the undifferentiated subtype. All of the enrolled subjects were right‐handed, and they had normal vision, normal structural magnetic resonance imaging (MRI), and no other significant medical or neurological disorders. We confirmed the diagnoses of schizophrenia with the Structural Clinical Interview for the DSM‐IV (First, Gibbon, Spitzer, & Williams, 1996). This study was performed according to the guidelines established by the local Institutional Review Board, and written informed consent was obtained from each participant. The demographic and clinical information of the subjects are summarized in Table 1.

**Table 1:** Demographic and clinical data of subjects

	Patients (*n* = 16)	Controls (*n* = 16)	*t*	*p* value
Male/female	8/8	8/8		
Age	29.8 ± 5.3	30.7 ± 2.9	0.58	.570
Years of education	13.4 ± 2.4	15.8 ± 1.8	3.22	.003
K‐WAIS	96.7 ± 12.9	109.1 ± 6.2	3.46	.002
PANSS positive	17.6 ± 8.9			
PANSS negative	17.7 ± 6.8			
PANSS general	37.8 ± 13.2			
Duration of illness (years)	6.6 ± 6.2			
Chlorpromazine‐equivalent dose for antipsychotic drugs (mg)	311.1 ± 226.6			

K‐WAIS, Korean‐Wechsler Adult Intelligence Scale; PANSS, Positive and Negative Syndrome Scale.

### 2.2 Behavioral tasks

**Figure 1:**
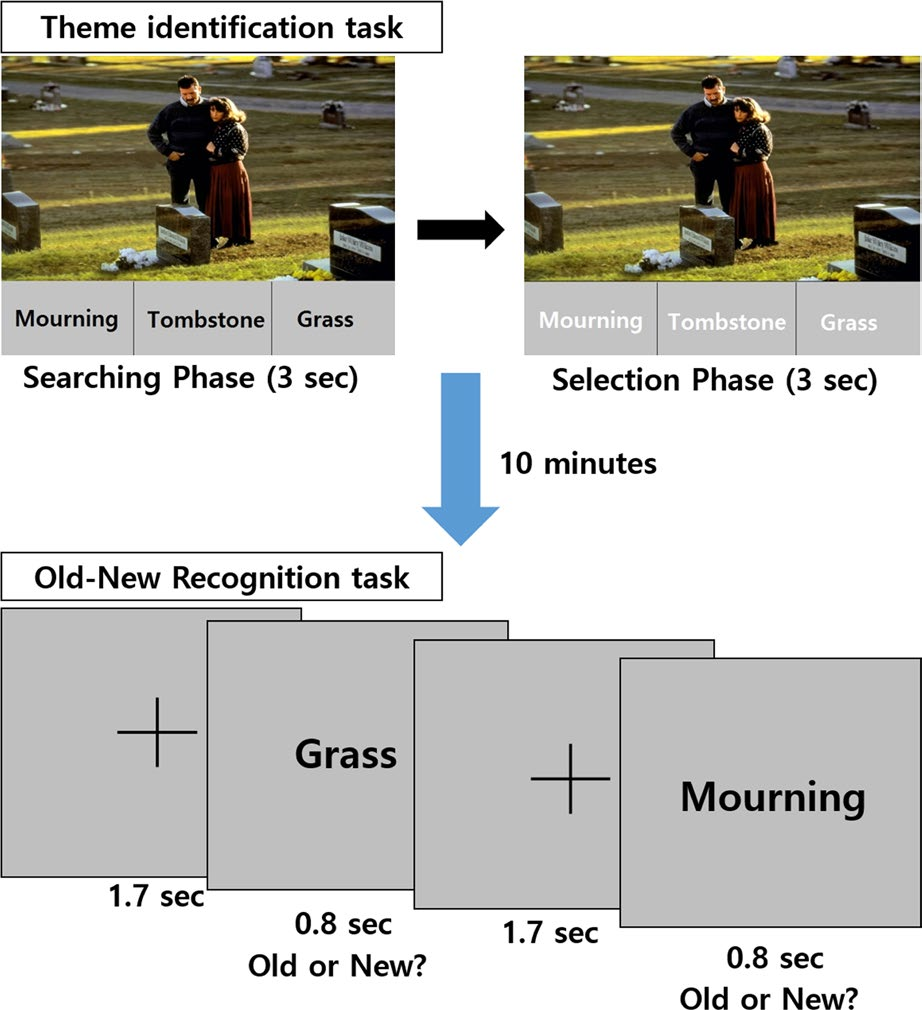
The sequence of task: theme identification and old–new recognition

The behavioral tasks consisted of the theme‐identification task, emotion‐selection task, and old–new recognition task, and they were conducted sequentially. In the functional MRI (fMRI) scanner, the participants first performed the theme‐identification task as an encoding task (Figure 1). Emotive pictures from the International Affective Picture System (Lang, Bradley, & Cuthbert, 1995) were presented with three words: a theme word, which was the main theme word; a related word, which was related to the theme but more concrete than the theme word; and an unrelated word, which was present in the stimulus picture but which did not have a direct relationship with the main theme. The subjects were asked to select the theme word for each picture among the three words. A total of 40 emotive pictures and, accordingly, 120 words were presented in an event‐related design. The study participants were not explicitly requested to memorize the words during the session. The 40 pictures consisted of 20 images of positive emotions and 20 images of negative emotions. The images were chosen based on the results of a pilot experiment of 16 normal participants (eight males who were not the same subjects as those included in the main experiment). In the pilot experiment, the subjects were asked to rate 120 pictures from −2 (very negative emotion) to 2 (very positive emotion). Finally, the 20 most positive and 20 most negative images were chosen for the main experiment.

After the theme‐identification task, the subjects performed the emotion‐selection task while they were still in the fMRI scanner. In this task, the subjects were asked to rate the emotions of the same 40 pictures that were used in the theme‐identification task as positive, neutral, or negative. More detailed information on the theme‐identification and emotion‐selection tasks, including the stimulus duration and jittered interval, has been previously presented (Oh et al., 2015).

After the emotion‐selection task, the subjects performed the old–new recognition task in the fMRI scanner (Figure 1). This task also utilized an event‐related design. The presented stimuli consisted of 160 words, with 120 being old words that were used in the theme‐identification task and 40 being new words. All of the words that were used in the theme‐identification task were used as old words. Therefore, the 120 old words included words from all three word categories (theme word, related word, and unrelated word). The new words consisted of 20 abstract words, such as health and start, and 20 concrete words, such as pencil and paper. The theme words were relatively abstract, while the unrelated words were relatively concrete in the theme‐identification task. In this session, the subjects were asked to discriminate whether the presented words were old or new. Each word was shown to the participants for 800 ms, after which they were required to select old or new during the subsequent 1,700 ms. Therefore, each word was presented for a total of 2,500 ms. In addition, we intermittently included null events with durations that ranged from 2,500 to 5,000 ms. Thus, the intervals between the two stimuli varied between 2,500 to 7,500 ms. Taken together, the total duration of the experiment was approximately 8 min. The sequence of pictures was randomized, and we jittered the interval between the trials with the Optseq2 program (http://surfer.nmr.mgh.harvard.edu/optseq/, RRID:SCR_014363).

### 2.3 Image acquisition

The MRI scanning was conducted with a 3T Philips scanner (Achieva; Philips Healthcare, Best, the Netherlands). Thirty‐six contiguous 3.8‐mm‐thick axial slices were collected with a single‐shot, echo planar imaging sequence depicting the blood‐oxygen‐level‐dependent signals (echo time = 40 ms; repetition time = 2,500 ms; flip angle = 90°; field of view = 220 mm; and image matrix = 128 × 128). T1‐weighted axial images (echo time = 4.6 ms; repetition time = 9.703 ms; flip angle = 30°; field of view = 220 mm; and image matrix = 256 × 256) that were 1.2‐mm‐thick were also collected.

### 2.4 Behavioral data analysis

The behavioral performances on the old–new recognition task were analyzed with a repeated‐measures analysis of variance with group (patients or controls) used as the between‐subjects factor and condition (theme words, related words, or unrelated words) used as the within‐subject factor. We also compared the group differences with post hoc *t* tests. We measured the discriminability and response bias in the old–new recognition task with the two‐high threshold theory (Snodgrass & Corwin, 1988). Discriminability, which demonstrated the ability of the subjects to discriminate old words from new, was defined as the percentage of correct recognition of old words minus the percentage of false recognition of new words. The response bias, which reflected the subject's tendency to falsely recognize new words, was defined as follows:

$$\text{Response bias}=\frac{\text{FA}}{1-(H-\text{FA})},$$

where *H* is the hit percentage and FA is the false alarm percentage. Response biases greater than 0.5 were considered liberal biases, and these indicated that the subject tended to say yes when he/she was unsure. In contrast, biases less than 0.5 were classified as conservative biases, and these indicated that the subject tended to say no when he/she was unsure. The statistical analyses were conducted with SPSS 20 (RRID:SCR_002865; IBM Corporation, Armonk, NY, USA).

### 2.5 Neuroimaging data analysis

A neuroimaging data analysis was performed on the fMRI data that were collected during the old–new recognition task. Preprocessing was conducted with FSL (FMRIB Software Library) version 5.0.8 (www.fmrib.ox.ac.uk/fsl, RRID:SCR_002823). The first three volumes were discarded to allow for signal equilibration. Motion correction was performed with FMRIB's Linear Image Registration Tool (MCFLIRT; Jenkinson, Bannister, Brady, & Smith, 2002), with a slice‐timing correction and high‐pass filtering cutoff point of 100 s in order to remove irrelevant signals. The BET brain extraction tool (Smith, 2002) was utilized to eliminate non‐brain voxels in the data. We used a 5‐mm full‐width at half‐minimum Gaussian kernel for smoothing. Registration to the T1‐weighted image of the corresponding subject and Montreal Neurological Institute space was conducted with FLIRT (Jenkinson et al., 2002).

The brain activations that were detected in the two groups in each condition were compared with FEAT version 6.00 (FMRI Expert Analysis Tool) in the FSL program. During the first‐level analysis, individual contrast maps were generated by contrasting the theme‐minus‐new condition, related‐minus‐new condition, and unrelated‐minus‐new condition. Two‐sample *t* tests of the three conditions were performed during the second‐level analysis of the correctly answered events. *Z*‐statistic images were thresholded with clusters that were determined by *Z* > 3.1 and a corrected cluster significance threshold of *p *=* *.05.

A functional connectivity analysis was conducted with an independent component analysis (ICA). FSL Multivariate Exploratory Linear Optimized Decomposition into Independent Components (MELODIC) version 3.14 was utilized to perform a probabilistic ICA (PICA; Beckmann & Smith, 2004). A PICA was applied because the sequences in which we presented the stimuli were not the same for each subject. Therefore, the underlying time courses differed across the subjects. In other words, this analysis was intended to find the overall connectivity patterns during the entire old–new recognition task. To perform a PICA in the group analysis setting, we used a multisession temporal concatenation tool in MELODIC. The number of independent components was automatically calculated by the software, and 36 independent component maps were finally generated. The use of a relatively higher model order (>30) of ICA introduces subnetwork delineation at a more detailed level that separates noise more appropriately. Thus, a single brain region that is detected with an ICA can represent a subnetwork (Abou‐Elseoud et al., 2010; Rytty et al., 2013). A spatial ICA that used these 36 components was applied to find Task‐Related Networks in both groups. Variance normalization was also used. The independent component maps were thresholded with an alternative hypothesis test that was based on the fitting of a Gaussian/gamma mixture model to the distributions of the voxel intensities within the spatial maps and controlling the local false‐discovery rate at *p *<* *.5 (Beckmann, DeLuca, Devlin, & Smith, 2005; Beckmann & Smith, 2004). Among the 36 independent components, 13 were found to be artifacts or noises upon visual inspection. Thus, 23 components were identified as anatomically and functionally meaningful networks, which was in accordance with previously reported networks (Aboitiz, Ossandon, Zamorano, Palma, & Carrasco, 2014; Green, Kraemer, Fugelsang, Gray, & Dunbar, 2010; Kahnt, Chang, Park, Heinzle, & Haynes, 2012; Kotz, Cappa, von Cramon, & Friederici, 2002; Mohanty et al., 2007; Nekovarova, Fajnerova, Horacek, & Spaniel, 2014; Nocchi et al., 2012; Rosazza et al., 2014; Spreng, Sepulcre, Turner, Stevens, & Schacter, 2012; Turken & Dronkers, 2011; Uddin, Clare Kelly, Biswal, Xavier Castellanos, & Milham, 2009; Wu, Liu, Hallett, Zheng, & Chan, 2013).

To investigate the differences in the functional connectivity in each network between the groups, the FSL dual regression technique (Filippini et al., 2009; Littow et al., 2010; Veer et al., 2010), which permits voxel‐wise comparisons, was used. During this analysis, we first used the group ICA spatial maps in a linear model that was fit against the separate fMRI data sets and then obtained the time‐course matrices depicting the temporal dynamics for each subject and component. We were then able to estimate the subject‐specific spatial maps with the time‐course matrices. The component maps were collected across the participants into single four‐dimensional files (one file per original ICA map). In addition, FSL randomized nonparametric permutation testing was conducted with 5,000 permutations and a threshold‐free cluster‐enhanced technique (Nichols & Holmes, 2002) to control for multiple comparisons and evaluate the voxel‐wise significant differences between the groups. Significant differences between the groups were defined by *p* values less than .05 for the voxel‐wise changes in the FSL randomization tool. Subsequently, Bonferroni corrections were performed to account for the multiple comparisons conducted between the 23 independent components.

## 3 Results

### 3.1 Behavioral task

In the old–new recognition task, the patients exhibited a lower (*t *=* *4.17, *p *<* *.001) discriminability score (0.21 ± 0.19) compared with the controls (0.47 ± 0.16). However, for response bias, the two groups did not differ (patients: 0.34 ± 0.19, controls: 0.35 ± 0.15). The difference in discriminability between the groups was still significant after we controlled for the covariates of years of education and IQ (*t *=* *2.45, *p *=* *.021). The main effects of group (*F *=* *19.00, *p *<* *.001) and condition (*F *=* *63.73, *p *<* *.001) on the recognition percentages were significant, whereas the interaction effect of group × condition (*F *=* *0.52, *p *=* *.600) was not significant. The recognition percentages (%) of the theme words (patients: 62.25 ± 14.25, controls: 82.15 ± 5.43, *t *=* *5.05, *p *<* *.001) and related words (patients: 54.73 ± 11.13, controls: 72.40 ± 10.79, *t *=* *4.41, *p *<* *.001) differed significantly between the groups. Although the *t* value was much less than those obtained for the theme words and related words, the two groups differed significantly in the recognition percentages of the unrelated words (patients: 34.49 ± 21.81, controls: 48.77 ± 15.05, *t *=* *2.09, *p *=* *.046).

### 3.2 Neuroimaging data

**Figure 2:**
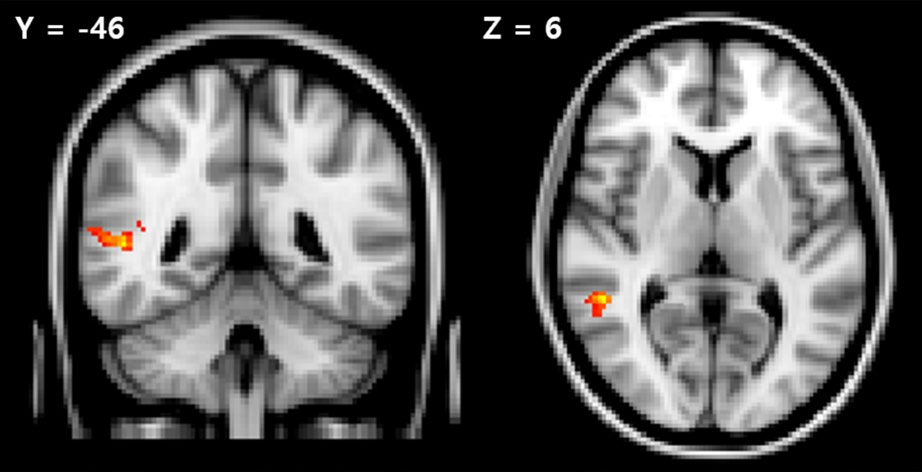
Brain regions showing significant group differences in the “theme‐minus‐new” contrast. Patients showed significantly increased activity compared with controls in the right superior temporal gyrus (185 voxels) during the correctly answered events. (FWE corrected *p *<* *.05, 3.1 [red] < *Z*‐score < 5 [yellow])

During correct responses, the patients showed significantly increased activity in the right superior temporal gyrus compared with the activity of the controls in the theme‐minus‐new contrast, while the other contrasts did not differ significantly (Figure 2). We performed a partial correlation analysis on all of the participants in order to detect any correlations between the activity of the right superior temporal gyrus in the theme‐minus‐new contrast and the ability to recognize theme words after eliminating the effects of the covariates of IQ and years of education. As a result, we found a significant negative correlation (*r *=* *−.48, *p *=* *.010) between the activity of the right superior temporal gyrus and the ability to recognize the theme words.

**Figure 3:**
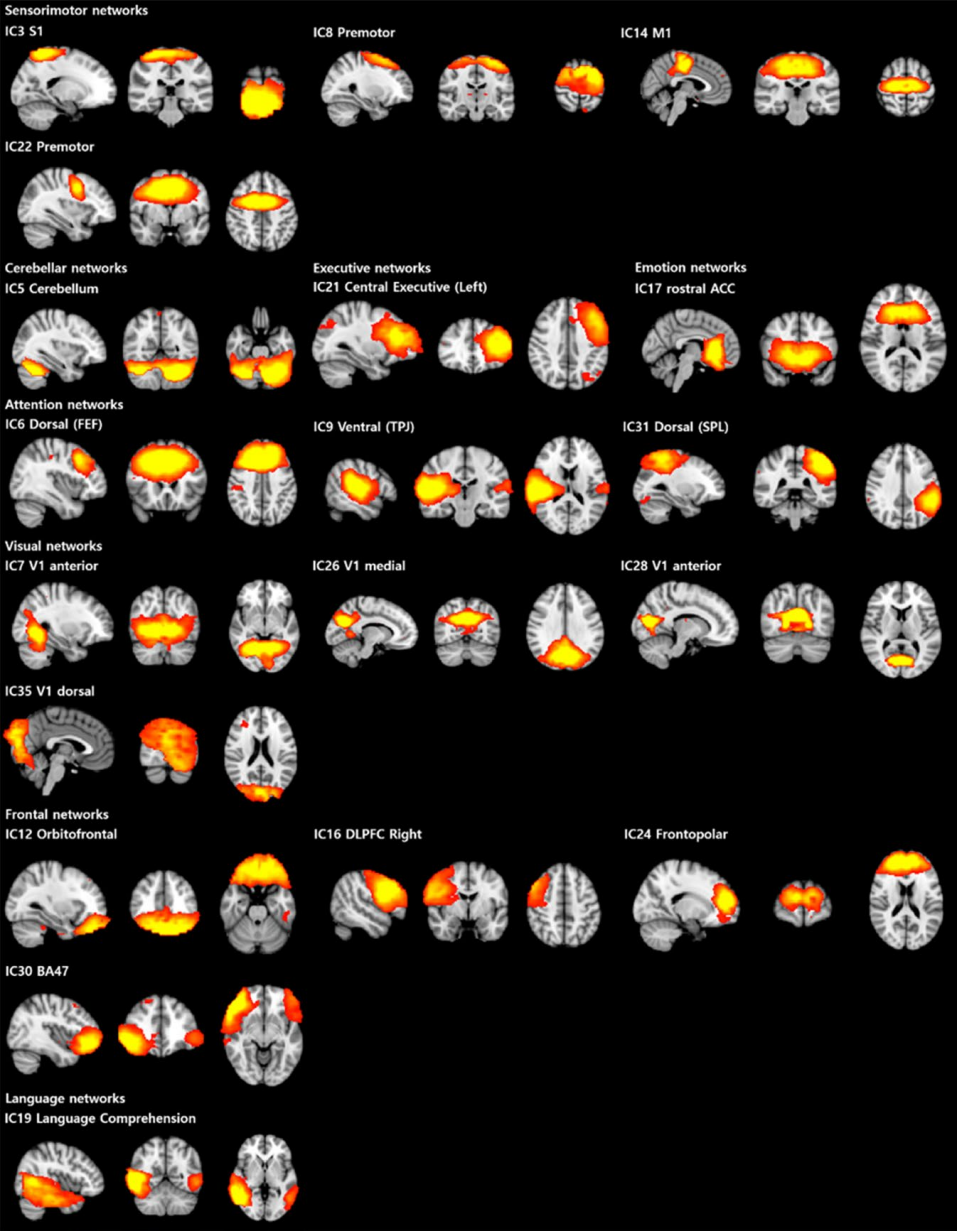
The 19 identified independent components for dual regression analysis. (The networks were shown using a 3 < *Z*‐score < 9 threshold). IC, independent component; S1, primary somatosensory cortex; M1, primary motor cortex; ACC, anterior cingulate cortex; FEF, frontal eye field; TPJ, temporoparietal junction; SPL, superior parietal lobule; V1, primary visual cortex; DLPFC, dorsolateral prefrontal cortex; BA, Brodmann area

**Figure 4:**
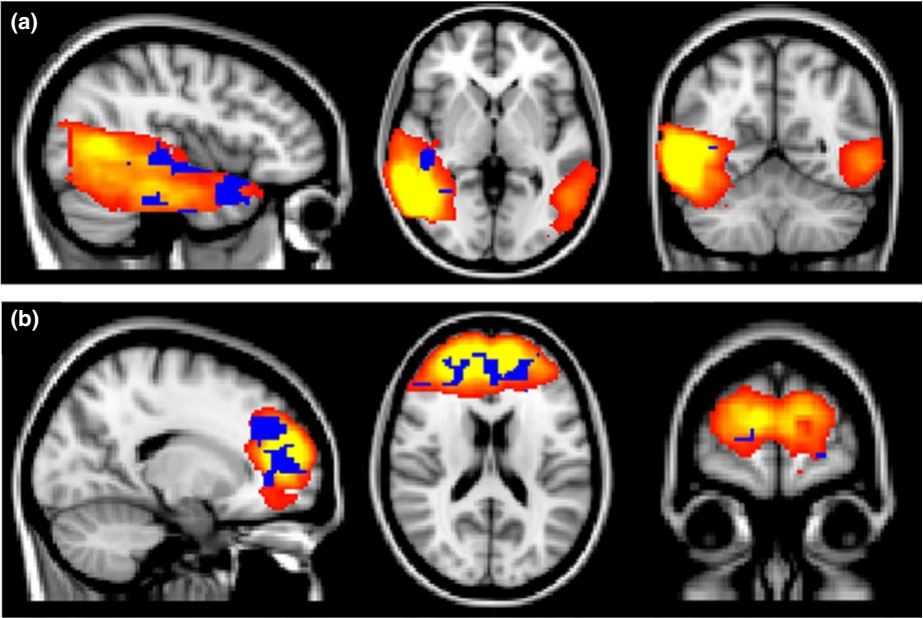
Brain networks showing significant group differences in the independent component analysis. Controls exhibited significantly increased connectivity compared with patients in these networks during old–new recognition (a: language comprehension networks, b: frontopolar networks). Red: 3 < *Z*‐score < 9: Yellow. Blue: Voxel‐level corrected *p *<* *.05

Twenty‐three independent components were initially identified as meaningful networks. We found that four of them were related to default mode networks, which left 19 components that were identified as Task‐Related Networks. The 19 components are depicted in Figure 3. Among the 23 independent components identified, the patients exhibited significantly decreased activity in five components compared to controls and significantly increased activity in two components after voxel‐level correction, as shown in Table 2. Bonferroni corrections were applied to correct for the multiple comparisons that were made across the 23 independent components, which resulted in two components demonstrating significant differences between the groups. In addition, these two components were involved with the decreased functional connectivity in the patients in the frontopolar and language comprehension networks (Figure 4).

**Table 2:** Functional brain regions showing significant group differences in functional connectivity during old–new recognition

IC	Regions	*N*_vox_	*Z*_max_	MNI coordinates		
				Max		
				*x*	*y*	*z*
Control > schizophrenia						
IC12	Frontal network (orbitofrontal)	201	4.25	18	46	−12
**IC19**	**Temporal network (language comprehension)**	**798**	**4.52**	**46**	−**32**	**0**
IC21	Executive network (central executive)	27	3.84	50	38	12
**IC24**	**Frontal network (frontopolar)**	**821**	**4.83**	**22**	**40**	**32**
IC25	Default mode network (PCC)	20	4.29	−6	−20	44
Schizophrenia > control						
IC14	Sensorimotor network (M1)	2	4.55	38	16	56
IC33	Default mode network (PCC)	16	3.94	22	−50	12

IC19 and IC24 still showed significant differences after Bonferroni correction for multiple comparisons across 23 independent components

MNI, Montreal Neurological Institute; *N*_vox_, number of voxels.

## 4 Discussion

The old–new recognition task yielded significantly lower discriminability scores for the patients compared with the controls, while the two groups showed similar response biases. In other words, the overall performance of the patients was inferior to that of the controls. With the exception of the new words, the patients did not recognize the theme words, related words, or unrelated words as well as the controls did. These results indicated that the patients with schizophrenia could not remember the old words like the healthy controls did, even if the information was theme‐related. Our results showed some trends. First, the patients exhibited more significant deficits in recognition memory when they had to recognize the words that were relevant to the theme of the image that had been previously presented.

Considering that the encoded words were presented with pictures depicting people's behaviors or appearances and that the presented words were related to those pictures, our results suggested that the patients were unable to memorize or utilize the information that was related to the people. Generally, in social situations, people can adjust their behavior according to their experiences. In other words, an exact understanding of one's own abilities, attitudes, and traits from experiences can help a person to know their role in society and compare themselves to other people (Harvey, Lee, Horan, Ochsner, & Green, 2011). Given that the patients exhibited more significant differences for the recognition of more important words compared with the controls, the patients might have some difficulties in learning and modifying their behavior according to key information gleaned from daily life. Therefore, problems with recognition memory in patients with schizophrenia can lead to social dysfunction in the patients. However, deficits in recognition memory that were related to nonsocial contexts were not directly compared in this study. Some studies have reported evidence of deficits in recognition memory that is related to nonsocial contexts as well (Kim, Kwon, Kang, Youn, & Kang, 2004; Weiss et al., 2009). Therefore, this viewpoint should be supported by the results of a task involving both social and nonsocial contexts, which should be able to determine whether the deficits were prominent during social contexts.

In order to elucidate the neural correlates of the deficits in recognition memory in the patients with schizophrenia, we first analyzed the fMRI data with two‐sample *t* tests. Only the correctly answered events were used in these analyses so that we could investigate the differences in neural activation between the groups and show similar recognition abilities. As hypothesized, during the correctly answered events, the patients exhibited increased activity in the right superior temporal gyrus compared with the controls in the theme‐minus‐new contrasts. These results might be explained by compensatory activity of the temporal lobe in the patients during the memory process, as has been suggested by previous research (Achim & Lepage, 2005; Ragland et al., 2004, 2009). Our results indicated that the patients required more neural activation, especially in the key information condition, in order to exhibit the same level of recognition performance as the controls. In addition, we found a negative correlation between the activity in the right superior temporal gyrus and the ability to recognize the theme word. Considering that patients with schizophrenia suffer from disconnection problems, these results suggested that overactivation was needed to compensate for the aberrant connectivity of this region and that the overactivation itself indicated low performance. Taken together, the well‐formed functional connectivity of the networks containing this area was important for answering correctly during the main‐theme‐word events in the old–new recognition task.

In the ICA connectivity analysis, the patients showed aberrant connectivity in the frontopolar and language comprehension networks during the old–new recognition task, and these results were in line with our initial hypothesis. The frontopolar region is responsible for abstract thinking (Azuar et al., 2014; Badre, 2008; Botvinick, 2008; Oh et al., 2015), and it plays a key role in the integration of information coming from multiple sensory systems in order to form a conceptual interpretation of the environment (Petrides & Pandya, 2006). Moreover, the frontopolar regions contribute to memory retrieval (Chua, Pergolizzi, & Weintraub, 2014; McDermott et al., 2000), and this recognition ability can be enhanced with the transcranial magnetic stimulation of these areas, as reported previously (Ryals, Rogers, Gross, Polnaszek, & Voss, 2015). Even if the patients successfully encoded the information during theme identification, the decreased network activity of the frontopolar region could lead to unsuccessful recognition.

In the language comprehension network, the patients showed significantly decreased connectivity, which was particularly prominent in the right hemisphere. These results supported our opinion that the compensatory activity in the right superior temporal lobe in the patients indicated the decreased connectivity of that region. Generally, a dominance of the left hemisphere in language‐related areas was observed, which was in accordance with the results of previous studies of healthy controls only (Galaburda, LeMay, Kemper, & Geschwind, 1978) or healthy controls and patients with language disorders (Herbert et al., 2005). However, other studies of healthy controls have reported results that demonstrate right lateralization of the language comprehension network, even though the motor speech network is still lateralized to the left (Zhu et al., 2014). In particular, this rightward asymmetry has been shown when healthy subjects are processing figurative language or comprehending someone's communicative intent in studies using healthy controls only (Proverbio, Crotti, Zani, & Adorni, 2009) and both healthy controls and patients with schizophrenia (Mitchell & Crow, 2005). Given that the main theme word did not indicate the immediate‐specific stimulus or situation but rather explained the situations in more general and symbolic terms, many of our stimuli in the old–new recognition task could be considered figurative. Furthermore, our stimuli were related to social situations. Thus, the words we presented had some communicative features. Considering that the patients demonstrated larger deficits in recognizing the main theme words, figurative features, and features related to the social situations of the main theme word could affect the rightward lateralization of the decreased network activity in the patients. In addition, the evidence that the rightward lateralization might be related to the emotional contexts of our data has been reported (Craig, 2011). According to that previous study, highly emotive situations can affect rightward lateralization, including the insula. Because our stimuli were relatively related to highly emotive situations, they could have affected the rightward lateralization of the decreased network activity in the patients.

Our results suggested that people have to utilize the frontopolar and language comprehension networks to successively recognize the key social information in daily life and that the patients with schizophrenia were not able to use these networks effectively. Although determining which network is more important is difficult, the proper comprehension of the surrounding environment or situation and the effective integration of the information are both essential components for remembering daily experiences and performing appropriate social roles.

Several important questions remain due to the limitations of our study, and they should be addressed in future studies. First, our sample size was relatively small. Second, the groups differed significantly in years of education and IQ. However, the discriminability still differed significantly between the groups, even after we controlled for the covariates of years of education and IQ. Another limitation of this study was that the patient group consisted of only medicated patients. Thus, treatment‐naïve patients with schizophrenia should be examined. In addition, we examined the overall connectivity pattern during the recognition process instead of elucidating the connectivity during specific conditions because the sequences in which the stimuli were presented differed for every subject. We were therefore unable to apply other ICA methods, such as Tensor‐ICA (Beckmann & Smith, 2005).

The group comparisons of the fMRI data revealed that the patients with schizophrenia demonstrated increased and compensatory activity in the right superior temporal gyrus so that they could exhibit similar levels of recognition performance of the socially relevant information as the healthy controls. A network analysis using the ICA method showed that the origin of this hyperactivity might be related to aberrant connectivity in the language comprehension networks. Furthermore, the abnormal connectivity in the frontopolar regions that was found could result in low recognition performance and social dysfunction in patients with schizophrenia. Therefore, the frontopolar and language comprehension networks could be potential therapeutic targets for patients with schizophrenia.

## Conflict of Interest

All authors declare that they have no conflicts of interest.
